# Preface: Recent Findings in Mercury Health Effects

**DOI:** 10.1289/ehp.8901

**Published:** 2006-01-18

**Authors:** Janice W. Yager, Milena Horvat

**Affiliations:** 1 Environment Division, Electric Power Research Institute (EPRI), Palo Alto, California USA; 2 Department of Environmental Sciences, Jožef Stefan Institute, Ljubljana, Slovenia

The four articles in this mini-monograph represent a cross-section of results from human studies on mercury exposure and health outcomes presented at the most recent International Conference on Mercury as a Global Pollutant (ICMGP) held 28 June to 2 July 2004 in Ljubljana, Slovenia.

The ICGMP provides a needed global forum for the exchange of innovative ideas, and it provides an opportunity to communicate research results to public policy makers, industry experts, and public representatives in order to promote the direct use of scientific and technical data to environmental protection and control. Intense interest in mercury is evident from the recommendations adopted by the European Union Mercury Strategy in June 2005 (European Union 2005); also, a Global Mercury Assessment was approved by the Governing Council of the United Nations Environmental Program in early 2002.

The 7th ICMGP was held in Ljubljana, Slovenia, the location of the second largest mercury mine in the world—the Idrija mercury mine. Although the mercury mine was the basis of prosperity of the town of Idrija for five centuries, it also caused extensive contamination of the town and its surroundings, leading to high levels of mercury exposure of the miners and the other inhabitants. The mercury mine was closed in the mid-1980s to avoid further releases of mercury to the environment.

Mercury research in Slovenia was initiated by the Jožef Stefan Institute in the early 1960s as a result of environmental and health concerns related to the operation of the mine. Initially, the main goal of the institute was to address the health status of the miners. Studies of the effects of mercury on the environment soon followed, and results have been published widely in the scientific literature.

The 7th ICMGP consisted of more than 600 scientific presentations, with nearly 500 participants from more than 40 countries, and covered a wide range of topics including analytical chemistry, biogeochemistry, atmospheric science, health effects, and remediation and policy issues. The conference was sponsored by a several Slovenian institutes, ministries, and businesses, including the Jožef Stefan Institute, Ministry of Education Science and Sport, Ministry of Health, Ministry of the Environment and Physical Planning, Thermo Power plant Šoštanj, Salonit Anhovo, Slovenian Tourist Board, Ljubljana Turist Board, Mercury Mine Idrija, Cankarjev Dom. International sponsors included Ina Naftaplin, the U.S. Environmental Protection Agency, the Electric Power Research Institute, Tetra Tech, Inc., Tekran Inc., Oak Ridge National Laboratory , Forschungszentrum Geesthacht GmbH (GKSSl), United Nations Environmental Program, Exponent, Global Environmental Facility/United Nations Development Programme/United National Industrial Development Organization (GEF/UNDP/UNIDO), (EUROCHLOR); National Institute for Minamata Disease, CEBAM Analytical Inc., Lumex, and International Research Development Center of Canada.

Information on the 8th International Conference on Mercury as Global Pollutant is available online (http://www.mercury2006.org).

Two studies in this mini-monograph address human exposure and outcomes related principally to elemental mercury vapors from past mercury mining operations (Kobal et al. 2006) and current high ambient exposures in China due to mercury mining, ore processing, coal combusion, and chloralkali plant operations (Chen et al. 2006) The other two studies address human exposure to methylmercury that occurred principally via fish consumption (Canual et al. 2006; Spurgeon 2006).

Together, these articles provide a cross-section of the highly active area of environmental health research regarding exposure to inorganic and organic mercury and health outcomes. Results of the studies provide counter-intuitive insights into the complexities regarding exposure and health effects. For example, although exposure to extremely high levels of mercury in the 18th century hat-making industry led to the “mad as a hatter” syndrome, relatively long-term, considerably lower exposure in the mercury mining industry does not appear to be directly associated with untoward personality traits. Investigation of interactions *in vivo* between selenium and mercury pointed to likely protective pathways in humans; this interaction has been previously predicted *in vitro.* Risk assessment methods generally assume that mercury hair levels directly reflect total mercury intake and most especially methylmercury intake from fish consumption. Results from the study of distinct populations in eastern Canada indicate that a considerable number of additional factors need to be determined and considered. Most important, these results imply that a direct linear relation between hair mercury and methylmercury intake cannot always be assumed. And finally, an evidence-based review of epidemiology studies of *in utero* methylmercury exposure and child developmental outcomes indicates that because of large uncertainties in both exposure assessment and outcome measures across studies, firm conclusions cannot be reached at this time as to whether there is or is not an effect on child neurobehavioral and developmental outcomes at relatively low levels of exposure.
